# Accidental Ingestion of Molar Band and Its Management: Maintenance Is Better than Management

**DOI:** 10.1155/2013/891304

**Published:** 2013-02-06

**Authors:** Appasaheb Naragond, Smitha Kenganal, K. Rajasigamani, N. Sathish Kumar

**Affiliations:** ^1^Department of Orthodontics and Dentofacial Orthopedics, P.M.N.M Dental College and Hospital, Bagalkot, Karnataka, India; ^2^Department of Conservative Dentistry and Endodontics, P.M.N.M Dental College and Hospital, Bagalkot, Karnataka, India; ^3^Department of Orthodontics and Dentofacial Orthopedics, Rajah Muthiah Dental College and Hospital, Chidambaram, Tamil Nadu, India; ^4^Sri Manakula Vinayagar Medical College and Hospital, Kalitheerthalkuppam, Puducherry, Tamil Nadu, India

## Abstract

Ingestion of a broken part of fixed orthodontic appliance is a potential complication during orthodontic treatment. We report a case of accidental ingestion of molar band and its subsequent diagnosis followed by endoscopic retrieval method. Although prevention of such incidence is the best method at the same time management of such an event is also crucial. The objective of this paper is to draw attention to the potentially serious complications that can occur if preventive techniques are not practiced and also the management of such event.

## 1. Introduction 

Management of orthodontic appliance without debonding or fracture is the biggest task for orthodontic patient during treatment. It requires ulmost care, cooperation, and maintenance. Accidental ingestion of orthodontic appliance or a part of it can cause severe problem in the airway or in gastrointestinal tract. Accidental ingestion of an appliance during a chair-side procedure or later, because of inadequate stability or retention of the appliance, can create a medical emergency that can lead to serious complications, like severe breathlessness or internal hemorrhage leading to death from aspiration of the foreign body. 

While a wide variety of complications resulting from foreign bodies have been documented in clinical practice, incidence of broken orthodontic appliances or components, dentures, and removable appliance are in majority.

 The incidence of aspiration or swallowing dental foreign bodies varies considerably in the literature. Ingestion or aspiration of foreign bodies is recognized as a complication in all clinical specialties of dentistry [1]. Accidental foreign body ingestion or aspiration is usually handled by physicians in the accident and emergency units. A sizeable proportion of those affected are children (80%) below 3 years of age [2]. While food materials constitute the majority of foreign bodies found in the airway in children [2], loose dentures, broken orthodontic appliances, or components and dental instruments are the second most commonly ingested objects in adults [3]. The incidence of aspiration or swallowing dental foreign bodies varies considerably in the literature, in a review article, the range was 3.6% to 27.7% of all foreign bodies, with the number considerably higher in adults than in children [4]. The aspiration or ingestion of orthodontic appliances is less common but not less varied in the types of appliance involved. These include swallowing an expansion appliance key [5], a transpalatal arch [6], a lower spring retainer [7], a fragment of a maxillary removable appliance [8], and a piece of archwire [1]. A rare case of accidental swallowing of a removable quad helix by a 13-year-old boy affected by Down's syndrome, which necessitated surgical removal, has also been reported [9]. A case of accidental ingestion of molar band and its subsequent diagnosis followed by endoscopic retrieval method is being presented. Guidelines for prevention and also management of such event are being discussed.

## 2. Case Presentation

**Figure 1:**
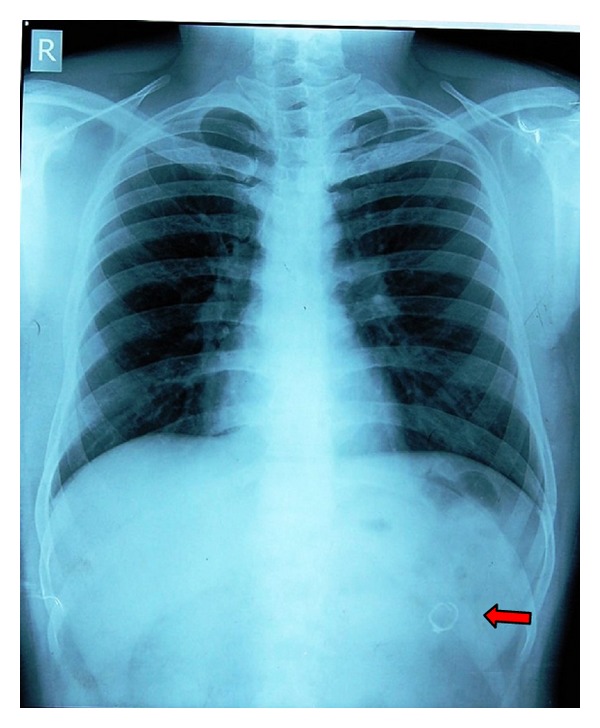
X-ray showing evidence of molar band.

A 16-year-old boy sought treatment with the chief complaint of proclined upper and lower anterior teeth. To fulfill the objectives of the patient we decided to go for fixed orthodontic appliance therapy along with extraction of all first premolars. All the first and second molars were banded. Upper and lower bonding was done. Patient's followup was done for three months. After that the patient reported to the clinic a complaint of ingestion of some part of the appliance. On examination it has been noticed that second molar band in the upper right side was missing. In search of missing band we did abdominal X-ray. The swallowed band was found in the upper abdomen (Figure 1) with no associated features of perforation or peritonitis. As the patient was asymptomatic we decided to manage him conservatively and use the wait and watch strategy. He was advised to soft diet and laxatives and was admitted for observation. His stools were checked after each act of defecation.

**Figure 2:**
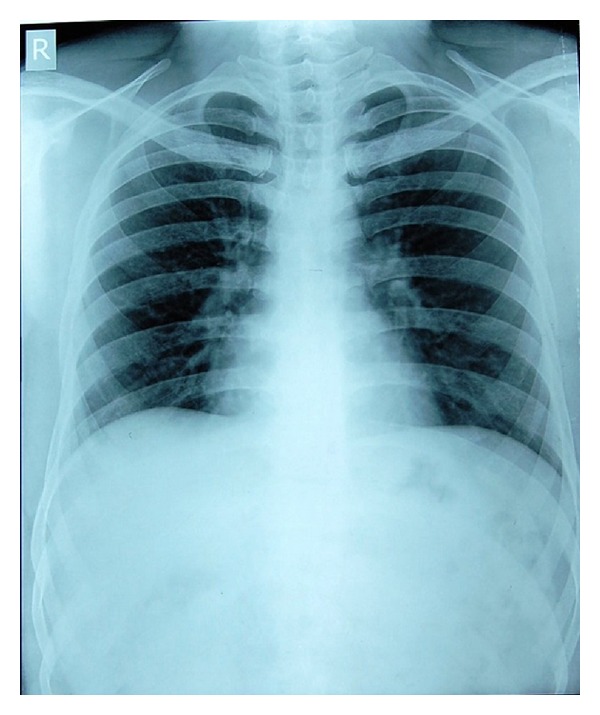
X-ray showing no evidence of molar band.

**Figure 3:**
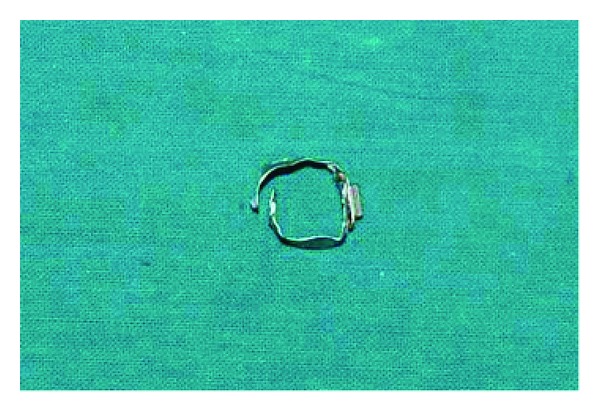
Retrieved molar band.

The patient was reevaluated after 72 hours; there had been no passage of molar band along with stool since admission. Repeat abdomen X-ray showed the molar band in the same position as it was in the previous X-ray; this was suggestive of impaction within the gastrointestinal tract. We decided to perform an endoscopy evaluation and attempt endoscopy retrieval of the molar band. At endoscopy it was noted to be in the distal part of stomach and molar tube hook was impacted in the mucosa. Using endoscopic grasper, the molar band was grasped, gently pulling it out of the gastric mucosa fold. The molar band was retrieved along with endoscope (Figure 3). After retrieval of the molar band repeat X-ray was done to rule out bowel perforation and it was normal (Figure 2). He was discharged after one day.

## 3. Discussion

 This paper illustrates the dislodgement of fixed appliance if the patient's cooperation in maintaining the appliance and retention of the appliance is inadequate. Foreign body ingestion or aspiration has the potential to result in acute medical and life-threatening emergencies. Some complications that can arise are bronchial stenosis, bronchiectasis, lung abscess, tissue ulceration or erosion, esophageal perforation with secondary mediastinitis, pneumothorax, intestinal obstruction, perforation with subsequent abscess formation, and hemorrhage or fistula.

 Early location of an inhaled or ingested foreign body facilitates appropriate and timely treatment management and referral. When a foreign body passes into the gastrointestinal tract, clinical symptoms and signs should be monitored closely until it is excreted or removed. Removable appliance are not the only orthodontic appliance that have given rise to problems. The problem was derived from so many fixed appliances also. Following precautions and recommendations are the outcome of this paper and other authors.

 An endodontic file can pass through the gastrointestinal tract asymptomatically and apparently atraumatically within 3 days [10]. When cutting the ends of arch wires with safety distal end wire cutters, the pliers sometimes fails to hold the cut fragment. A cotton wool roll placed over the end of the arch-wire before it is cut will prevent the piece of arch wire becoming displaced in the mouth, or embedded in the soft tissues of the patient or operator [11]. The use of gauze dental napkin as barrier technique can be very successful, when placed behind the orthodontic appliances during adjustment and cutting the distal edge [12]. In some patients, placing second molar bands is difficult. To keep control of bands in case of accidental slip to aid quick retrieval, floss can be tied through the tube. Once the band is cemented on the tooth, the floss can be removed. A similar technique has been recommended for the placement of rubber dam clamps [13].

 All the components of removable appliances should be smooth and rounded as far as possible. All cribs and springs should not have sharp ends, and finger springs and stops should be rounded [14]. Keys for turning fixed expansion appliances intraorally should be attached to floss and any open contact on the handle of the key should be soldered to prevent the floss from slipping through the handle [15]. When fitting or removing transpalatal arches and quadhelices, it may be advisable to have a long length of floss tied to the appliance attached via a closed loop on the appliance to avoid its inhalation or swallowing should it be dropped [16]. The choice of archwire material and grade should not only be dictated by the forces required to move teeth, but also by the ability of the wire to withstand masticatory stresses [6]. If a piece of appliance is dropped in the mouth during treatment, the availability of high-speed suction with a pharyngeal tip can help with quick retrieval [17]. The ideal method to locate a swallowed object is by X-ray evaluation. An X-ray helps to localize the site, show evidence of obstruction, onword progression and confirms the passage of swallowed object [1, 3, 10]. Endoscopic removal of foreign bodies is a safe and effective mode of management of swallowed object [1]. Failure at endoscopic retrieval is an indication for proceeding to surgical removal either by open or laparoscopic technique. The use of laxative is not a proven advantage and may increase the chance of perforation [18].

## 4. Conclusion

 Efficiency of the appliance to withstand the masticatory force and retention should be evaluated before delivering. An orthodontic appliance can also be dislodged by patient's negligence to maintain the appliance so it is important to educate the patient to maintain the appliance and mismanagement and also to early report to the doctor when such incidence occurs. Early location of an inhaled or ingested foreign body facilitates appropriate and timely treatment management and referral. It is very important to instruct and educate verbally and in written format regarding management, precautions, adverse effects of loose and broken appliance. When a foreign body passes into the gastrointestinal tract, clinical symptoms and signs should be monitored closely until it is excreted or removed. Noninvasive procedures for managing airway obstruction include the advising laxatives then wait and watch, Heimlich maneuver, abdominal or chest thrusts in pregnant or obese patients, and finger sweeps when the object is located in the oral cavity in unconscious adults. Foreign bodies lodged in the esophagus should be removed endoscopically, but some small, blunt objects may be pulled out using a Foley's catheter and the last option will be surgical removal either by laparoscopy or open technique.
